# Anti-immunoglobulin-like transcript 3 induced acute myocarditis—A case report

**DOI:** 10.3389/fcvm.2022.1035569

**Published:** 2022-12-08

**Authors:** Osnat Itzhaki Ben Zadok, Arthur Shiyovich, Ashraf Hamdan, Moshe Yeshurun, Inbar Nardi Agmon, Pia Raanani, Ran Kornowski, Liat Shargian

**Affiliations:** ^1^Department of Cardiology, Rabin Medical Center, Petah Tikva, Israel; ^2^Sackler Faculty of Medicine, Tel Aviv University, Tel Aviv, Israel; ^3^Davidoff Cancer Center, Rabin Medical Center, Petah Tikva, Israel

**Keywords:** case report, anti-ILT-3 induced myocarditis, immunotherapy, acute myocarditis, cardio-oncology

## Abstract

To the best of our knowledge, this is the first published report of anti-immunoglobulin-like transcript 3 (ILT3)-induced myocarditis. A 48-year old female patient with refractory acute myeloid leukemia who was given a single dose of anti-ILT3 monotherapy presented with fever, hypotension, chest pain, and elevated cardiac biomarkers. Systolic bi-ventricular function was in normal limits. The patient was promptly treated with pulse dose steroids with a rapid hemodynamic and clinical improvement and declining levels of cardiac biomarkers. The diagnosis of acute myocarditis was confirmed using cardiac magnetic resonance imaging applying the revised Lake Lewis criteria. While larger-scale data are needed in order to assess the incidence, management and prognosis of anti-ILT-3 induced myocarditis, we believe a high level of suspicion for adverse non-target cardiac effects is required in patients receiving this novel class of drugs.

## Introduction

A 48-year-old female patient, BRCA-1 carrier, was diagnosed with triple negative breast carcinoma on age 44 and treated with neoadjuvant chemotherapy (adriamycin, carboplatin, and paclitaxel) followed by bilateral mastectomy and radiotherapy. As part of a clinical research protocol, she was also given pembrolizumab, an immune checkpoint inhibitor (ICI), both as neoadjuvant and maintenance therapy for another 12-months. On age 47, the patient presented with tri-lineage cytopenia and was diagnosed with myelodysplastic syndrome with excess of blasts. Cytogenetics revealed a monosomal complex karyotype with deletion of chromosomes 5 and 7 with no molecular aberration. The patient underwent allogeneic hematopoietic cell transplantation from a matched sibling with myeloablative treosulfan-based conditioning, with full donor chimerism on day 30. Due to an increased risk for relapse, maintenance therapy with azacytidine and venetoclax was initiated on day 60, however on day 180 the patient relapsed with overt-transformation to acute myeloid leukemia. The patient was then treated with salvage chemotherapy (FLAG-IDA protocol) combined with venetoclax for 7 days, yet unfortunately she did not respond. Finally, the patient was recruited to a clinical immunotherapy trial in a different hospital.

**Figure 1:**
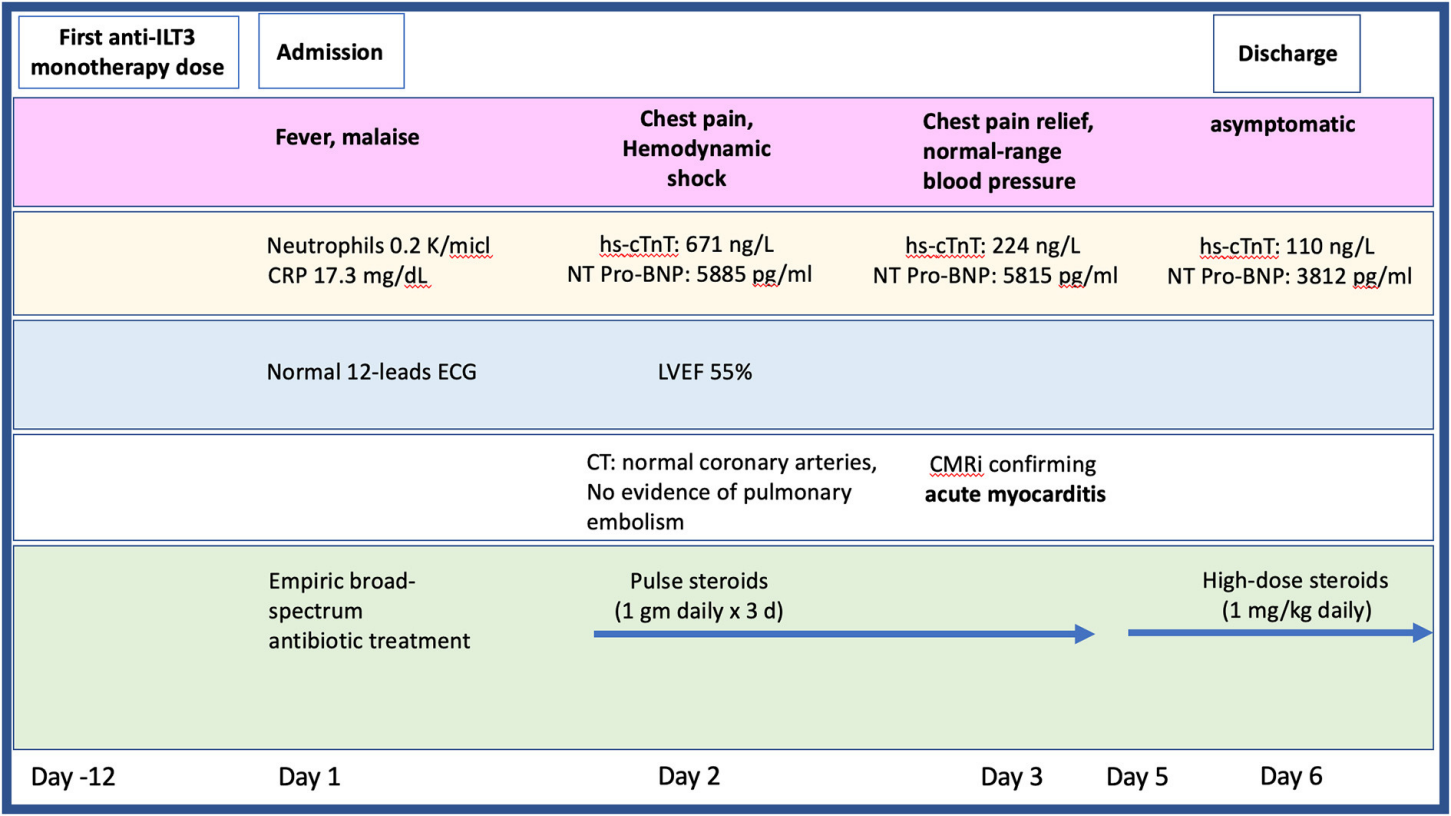
Timeline presenting the patient's clinical presentation, diagnostic findings, and treatment approach during hospitalization. CMR, cardiac magnetic resonance imaging; CT, computed tomography; ECG, electrocardiogram; hs-cTnT, high sensitive troponin; LVEF, left ventricular ejection fraction; NT pro-BNP, N-terminal pro-brain natriuretic peptide.

**Figure 2:**
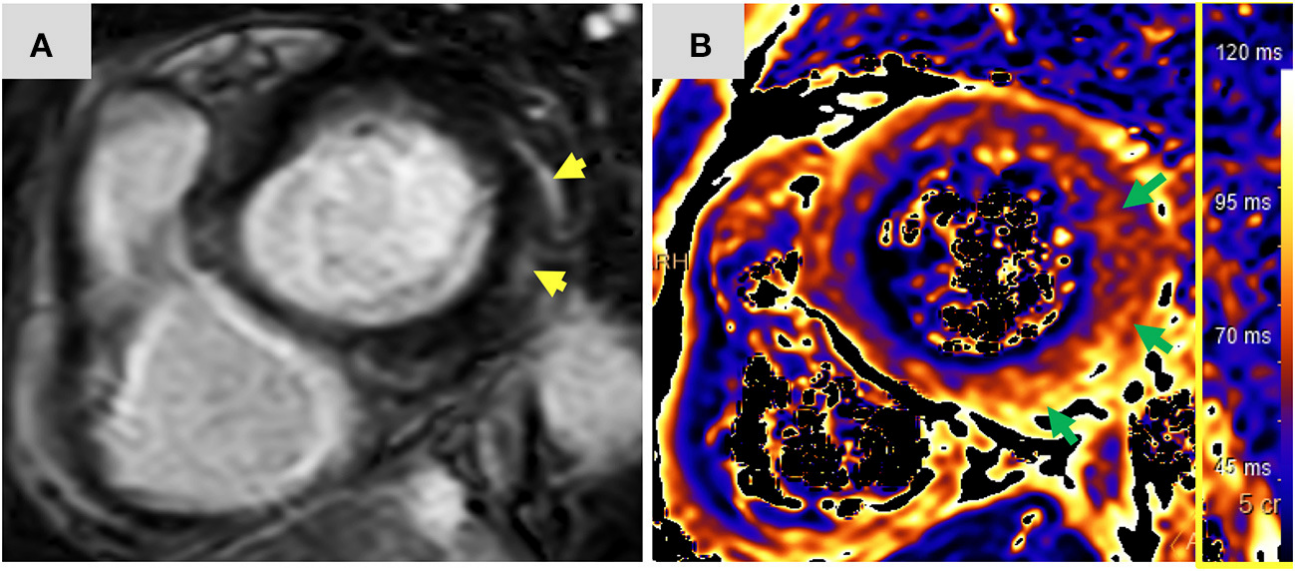
Magnetic resonance imaging findings: Short axis view **(A)** of late gadolinium enhancement illustrates sub-epicardial and mid-wall late gadolinium enhancement in the basal infero-lateral and antero-lateral segments (yellow arrows). The corresponding short axis view of T2 mapping **(B)** illustrates extensive diffuse interstitial myocardial edema (green arrows). Diffuse T2 value was 56.2 ms and focal T2 value in the infero-lateral segment was 61 ms (normal limit 55 ms). The presence of myocardial injury and extensive edema support the diagnosis of acute myocarditis according to the 2018 updated Lake Louise criteria. ms, miliseconds.

Twelve-days after her first monotherapy treatment with humanized IgG4 anti immunoglobulin-like transcript 3 (ILT3) [MK-0482, MERCK (MSD)] (75 mg), the patient presented to the Hemato-Oncology Ambulatory Care Unit in the Davidoff Cancer Center, Rabin Medical Center (Israel) with fever and malaise (patient's presentation and management time-line is presented in Figure 1). Physical examination was unremarkable and vital signs were in normal range except for systemic fever (temperature 101.3 °F (38.5°C). Laboratory analysis demonstrated 2.1 K/micl leukocytes (normal-range values 4.5–11 K/micl) with 0.2 K/micl neutrophils (normal-range values 1.8–7.7 K/micl), hemoglobin 8.5 g/dL and platelets 12 K/micl. C-reactive protein was 17.3 mg/dL (normal-range values 0.0–0.5 mg/dL). Chest x-ray was normal and 12-leads electrocardiogram (ECG) revealed sinus rhythm with a T-wave inversion in aVL. On a working diagnosis of neutropenic fever, blood cultures were collected and empirical antibiotic (Meropenem) was initiated. COVID-19 status was negative. On day 2, the patient began complaining of a constant chest pain which exacerbated with breathing and was not relieved by oral analgesics. She denied any shortness of breath, palpitations or muscle pain. Blood pressure was 83/52 mmHg, pulse 107/min, oxygen saturation was 96% on room air, respiratory rate 20 breaths per minute. Repeated ECG was unchanged. Lungs were clear to auscultation bilaterally and heart sounds were rapid with no apparent new murmurs. The patient did not show signs of volume overload or pulmonary congestion. Neurologic examination was unremarkable. Troponin T level and NT pro-BNP level were 671 ng/L (normal-range values 0–14 ng/L) and 5,885 pg/ml (normal-range values below 125 pg/ml), respectively. Echocardiography demonstrated a lower limit of normal left ventricular systolic function (LVEF 55%) similar to her prior routine echocardiogram study performed 3 months earlier. Given her clinical presentation and recent novel immunotherapy treatment, the leading diagnosis was immunotherapy-induced acute myocarditis, and decisions were made to monitor her in the cardiac unit and to immediately initiate therapy with empirical pulse dose steroids (methylprednisolone 1 g for 3 days). Cardiac computed tomography revealed normal coronary arteries and ruled out acute pulmonary embolism. On day 3, the patient reported an improvement in her wellbeing and the amelioration of her chest pain. Blood pressure stabilized (103/85 mmHg) and laboratory analysis showed declining levels of cardiac biomarkers (Troponin T 224 ng/L and NT pro-BNP 5,815 pg/ml). The diagnosis of acute myocarditis was confirmed using cardiac magnetic resonance imaging (Figure 2) based on the updated Lake Louis criteria demonstrating subepicardial and mid-wall late gadolinium enhancement in the basal infero- and antero-lateral segments with increased values of native T1 time and T2 time revealing extensive diffuse myocardial edema. After the completion of 3 days of pulse steroid therapy, the patient was switched to oral high-dose (1 mg/Kg) prednisone treatment. Elaborated diagnostic work-up for infectious etiology which included blood cultures, inspiratory viral PCR panel and bacterial, rickettsial and viral serologies was unrevealing.

The patient was discharged home after a 5-day hospitalization with a favorable functional status. Her Troponin T and NT-proBNP levels at discharge were 110 ng/l and 3,812 pg/ml, respectively. A decision was made to discontinue investigational drug therapy.

## Discussion

To the best of our knowledge, this is the first published report of anti-ILT3 monotherapy-induced acute myocarditis using the MK-0482 monoclonal antibody. ILT3, gene name LILRB4, is an inhibitory receptor expressed on myeloid-derived suppressor cells which is associated with immune tolerance within the tumor microenvironment. Anti-ILT3 were shown to abrogate myeloid immunosuppression and enable tumor killing (1). Moreover, antagonism of the ILT3 receptor may enhance the efficacy of immune checkpoint inhibitors (ICI) (2). Several phase 1 and phase 2 trials using these new class of drugs are currently ongoing worldwide in both solid and non-solid cancers. Data regarding both the efficiency and safety profile of these immunotherapy agents are still scarce, yet as recently presented in the American Society of Clinical Oncology annual meeting, the combined use of MK-0482 and pembrolizumab was associated with 2 cases of myositis, one of which was fatal (3). While cases of anti-ILT-3-induced myocarditis were not reported in this cohort, it is important to note that the co-existence of myositis and myocarditis is well-established with the use of ICIs (4). More data are needed in order to determine whether this clinical association also exists with the novel class of anti-ILT3 drugs.

Similarities between ICI-induced myocarditis and our presented case of anti-ILT-3 induced myocarditis are the short period of time between the first given dose of the antibody and the development of myocarditis [a median start of onset of 34 days of ICI-induced myocarditis (5–8)], the rise in troponin T which is evident in 94% of patients with ICI induced myocarditis (5, 9) and the presentation of acute myocarditis with normal left ventricular systolic function [found in 51% of patients with ICI-myocarditis (5–8)].

Due to the lack of published evidence regarding the treatment of anti-ILT-3-induced myocarditis and due to the similarities in the mechanism of action and clinical presentation to these two treatment modalities, we based our management strategy on current published scientific literature and society-guideline recommendations for ICI-induced myocarditis (8, 10, 11). Endomyocardial biopsy was not performed due to patient's severe thrombocytopenia, yet cardiac magnetic resonance imaging allowed for a definite diagnosis of acute myocarditis with extensive diffuse edema (12). The patient was hemodynamically monitored and cardiac biomarkers' levels were regularly followed. Prompt treatment (< 24 h from chest pain presentation) with high-dose steroids was given based on the recently published cardio-oncology society guidelines (8), as this was previously shown to improve patient's MACE and mortality (13). Due to patient's rapid hemodynamic improvement and declining biomarkers' levels, we did not use immunosuppressive drugs other than high-dose steroids, which provided both clinical and laboratory improvement. Notably, it is critical to underscore the importance of a good collaboration between the hemato-oncology and cardio-oncology providers, which allowed for the patient's rapid evaluation, diagnosis and therapy initiation.

While larger-scale data are needed in order to assess the incidence, management and prognosis of anti-ILT-3 induced myocarditis, we believe a high level of suspicion for adverse non-target cardiac effects is required in patients receiving this novel class of drugs.

## Data availability statement

The original contributions presented in the study are included in the article/supplementary material, further inquiries can be directed to osnatit@clalit.org.il.

## Ethics statement

Written informed consent was obtained from the participant for the publication of this case report and any potentially identifiable images or data included in this article.

## Author contributions

OI drafted the manuscript. AS, AH, MY, IN, PR, RK, and LS have reviewed and commented on the final draft. All authors contributed to the article and approved the submitted version.
